# The Effects of Precursor C_2_H_2_ Fraction on Microstructure and Properties of Amorphous Carbon Composite Films Containing Si and Ag Prepared by Magnetron Sputtering Deposition

**DOI:** 10.3390/nano9040528

**Published:** 2019-04-03

**Authors:** Xiaoqiang Liu, Junying Hao, Yongjun Lv, Xuejun Cui

**Affiliations:** 1 School of Metallurgy and Chemical Engineering, Jiangxi University of Science and Technology, Ganzhou 341000, China; 2 State Key Laboratory of Solid Lubrication, Lanzhou Institute of Chemical Physics, Chinese Academy of Sciences, Lanzhou 730000, China; jyhao@licp.cas.cn; 3 Material Corrosion and Protection Key Laboratory of Sichuan Province, School of Materials science and engineering, Sichuan University of Science & Engineering, Zigong 643000, China; yongjunlv@outlook.com (Y.L.); cxj_2046@163.com (X.C.)

**Keywords:** amorphous carbon, low friction, mechanical properties, nanoparticles, tribological behaviors

## Abstract

Hydrogenated graphite-like carbon composite films containing silicon (Si) and silver (Ag) (g-C:H:Si:Ag) were prepared by middle frequency magnetron sputtering deposition in argon (Ar) and acetylene (C_2_H_2_) mixture gases. The effects of precursor C_2_H_2_ fraction on the microstructure and properties were studied. The results of Raman and X-ray photoelectron spectroscope (XPS) revealed that the films were dominated by sp^2^ carbon sites. It was observed from transmission electron microscope (TEM) that the films contained nanoparticles mainly consisting of Ag, and their size increased with the decrease in the C_2_H_2_ fraction. Si was also found to aggregate in the areas where Ag nanoparticles formed in films with high Si content. The comparative studies on the frictional behaviors of films sliding against aluminum oxide were carried out in ambient air and saline solution. The g-C:H:Si:Ag films still exhibited outstanding frictional properties even when the test environment shifts from ambient air to saline solution.

## 1. Introduction

Amorphous carbon films have been drawing a lot of attention in recent years due to their high hardness, low friction, superior wear resistance, and good biocompatibility, and these properties make them possess potential widespread applications as protective films in the fields of engine parts and biomedical implants [1]. Nevertheless, the pure amorphous carbon films still suffer many disadvantages including high residual internal stress, low toughness, and poor adhesion strength, which greatly limit the wide application of the films [2].

So far, adding other elements such as Ti [3], Cr [4] or Mo [5] has been widely applied to overcome the above problems. Meanwhile, mechanical hardness and corrosion resistance [6] can also be improved by alloying these elements into the amorphous carbon films. Ti and W are widely applied to improve the hardness of amorphous carbon films due to the formation of TiC or WC carbides nanocrystalline. However, high content of such carbides can also lead to high brittleness and residual compressive stress. On the other hand, adding soft metals such as Al [7], Cu [8], and Ag [9,10,11]—known as weak-carbide-forming elements—has been extensively employed to reduce the residual stress of amorphous carbon films. However, such metal elements could also deteriorate the mechanical hardness.

Accordingly, the concept of duplex compositing has been developed to design superior amorphous carbon films with comprehensive properties based on the synergistic effect of composite elements on the microstructure and properties of films. The hard transition metals are commonly used to improve mechanical hardness, and weak-carbide-forming elements are employed to reduce residual stress and enhance the graphitization of sliding interface.

Different deposition techniques including chemical vapor deposition (CVD) and physical vapor deposition (PVD) have been employed to prepare the multi-composited amorphous carbon films. Typically, CVD is used to prepare non-metal composited films, such as the films containing sulfur and fluorine (a-C:S:F) [12] when the gaseous source of the introduced materials is applied. Metal composited amorphous carbon films are usually prepared by PVD techniques, including magnetron sputtering deposition, when the solid source of introduced materials was used. Results from Wang’s group [13,14] revealed that introducing Ti/Al can significantly reduce residual stress with no detriment to mechanical properties, and improve the tribological performance of diamond like carbon films. In addition, Zhou et al. [15] developed W/Al composite diamond-like amorphous carbon films with superior mechanical and tribological properties. Sui et al. [16] reported that the antifouling, anti-corrosion, and tribological properties of graphite-like carbon films can be comprehensively enhanced by the joint addition of Cr and Cu.

Furthermore, Si has been extensively alloyed into the amorphous carbon films to reduce their humidity sensitivity of the friction coefficient and enhance the low friction behaviors in the water solution because of silica hydrate formation in the sliding interfaces [17,18]. In our previous work, we found that the joint addition of Si/Al [19] relieved the humidity dependence of frictional performance of the hydrogenated amorphous carbon films, and achieved super-low friction in ambient air. Nevertheless, the environment suitability of amorphous carbon films’ tribological properties remain relatively weak and should be further improved to accommodate for the challenge of external environmental changes, i.e., amorphous carbon films should be designed with adaptive tribological performance in different environments.

In this work, amorphous carbon composite films containing Si and Ag were prepared based on the concept of duplex composite techniques. The influence of precursor C_2_H_2_ fraction on microstructure and properties of the composite films were investigated. The frictional behaviors of films in ambient air and saline solution were studied.

## 2. Experimental Details

### 2.1. Sample Preparations

The g-C:H:Si:Ag films were prepared by mid-frequency magnetron sputtering deposition on Si substrates in an Ar and C_2_H_2_ mixture atmosphere. It has been reported that the sputtering yield of Ag is ~7–8 times higher than that of Si when pure Ar ions are used [20]. Thus, in order to prepare the a-C:H:Si:Ag coatings with the content of Ag close to that of Si, we designed the rectangle-shaped target with ~ 19.2 cm^2^ area, consisting of Si (99.999 wt. %, Omat Group, Shenzhen, China) and Ag (99.99 wt. %, Omat Group, Shenzhen, China) with 1:8 area ratio. The mid-frequency direct current (DC) power (40 kHz) was charged on the twin-magnetron targets. The deposition chamber was evacuated to a base pressure of 2 × 10^−3^ Pa. Before the films’ deposition, the substrates were etched for 10 min by Ar ions, which were generated at a high negative bias voltage of −1000 V, and then an interlayer about 200 nm thickness was deposited through sputtering twin-targets in pure argon plasma. The flow rate ratio of C_2_H_2_ and Ar (C_2_H_2_/Ar) was tailored at the range from 1/6.0 to 1/7.5 to prepare the g-C:H:Si:Ag films with different contents of Si and Ag. The film deposition conditions are listed in Table 1.

### 2.2. Microstructure Characterizations

The film’s thickness was characterized on a surface profilometer. A PHI-5702 X-ray photoelectron spectroscope (XPS, Physical Electronics, Chanhassen, MN, USA) with monochromated Al Ka radiation was employed to detect the compositions of films operating at pass energy of 29.35 eV. The residual pressure of the system was ~10^−8^ Torr. The C1s XPS curves of the films were fitted through Gaussian distribution function after subtracted the Shirley background. The Raman spectra (HR800, Horiba Jobin Yvon, Longjumeau, France) of the films were obtained at a laser power of 0.1 mW operating with 532 nm Ar ion laser as the excitation source. The resolution of the spectra was ~2 cm^−1^. Gaussian fitting was used to the main Raman peaks of the samples to acquire the important parameters including peak positions and the intensity ratio of the fitted peaks. The fractured cross-sectional morphology of the films was observed by a field emission scanning electron microscope (FESEM, JSM 6701F, JEOL, Tokyo, Japan). The coated Si substrates were broken manually to prepare the cross-section observation by FESEM with the accelerating voltage of 5.0 kV under second electron image mode. A Tecnai G2 F20 transmission electron microscope (TEM, FEI, Hillsboro, OR, USA) with accelerating voltage of 200 kV was used to survey the nanostructure of the g-C:H:Si:Ag samples. The films with thickness of about 30 nm were deposited directly on the single-crystal NaCl substrates, which were dissolved in distilled water to collect sample fragments for TEM analyses. The linear distribution of the introduced elements was detected through the mode of high-angle annular dark field scanning transmission electron microscopy (HAADF-STEM) on TEM, and energy dispersive X-ray spectra (EDS) were acquired under the drift corrected spectrum profile mode.

### 2.3. Mechanical and Tribological Characterizations

A nano-indenter (TI950 Tribo-Indenter, Hysitron, Minneapolis, MN, USA) was applied to detect the elasticity, hardness, and elastic modulus of the deposited films. The maximum indentation depth was kept at ~10% of the film’s thickness to avoid the substrate’s influence. Five different points on each sample were selected to carry out the parallel nano-indentation tests. The elastic recovery rate was calculated based on the Equation (1), where *d_max_* and *d_res_* are the maximum and residual displacement, respectively.

(1) $$\mathrm{Elastic}\;\mathrm{recovery}\;\mathrm{rate}=\frac{\left(d_{max}-d_{res}\right)}{d_{max}}\times 100\%$$

The tribological performance of the samples was evaluated on a ball-on-disk tribometer (UMT-2MT, Center for Tribology, Inc., Campbell, CA, USA) in ambient air (Relative humidity, 30–45%) and in saline solution (NaCl, 0.9 wt. %) at the normal load of 3 N. Three tests were done on each sample under the same condition. The counterpart material was aluminum oxide (Al_2_O_3_, Shangtao Inc., Shanghai, China) ball (Diameter: 6.0 mm). The sliding velocity was set at 0.1 m·s^−1^. The lost volume of the wear track on the films was determined by non-contact method on Micro XAM-3D Surface Profile (ADE Phase Shift, Tucson, AZ, USA). The average wear rate was acquired according to the lost volume of the wear track on the films divided by the sliding distance and the applied load. The transfer layers on the counter balls were characterized by a scanning electron microscope (SEM, JSM-5600, JEOL, Tokyo, Japan).

## 3. Results and Discussion

### 3.1. Compositions and Microstructure

**Figure 1:**
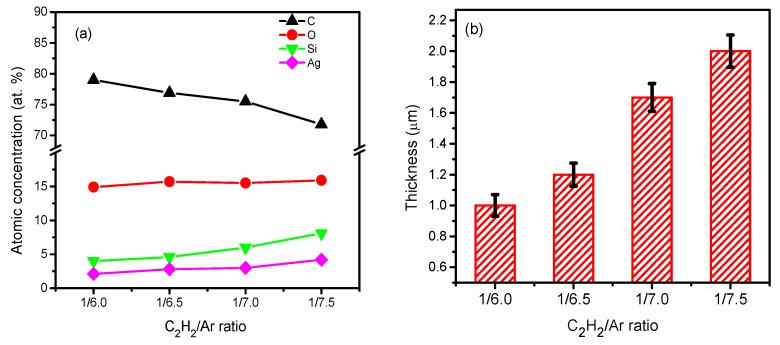
Composition (**a**) and thickness (**b**) of the films as a function of the C_2_H_2_/Ar ratio.

The chemical compositions of the g-C:H:Si:Ag films were found to relatively depend on the C_2_H_2_/Ar ratio. As demonstrated in Figure 1a, the contents of Si and Ag increased slightly while that of C decreased significantly with the decrease in C_2_H_2_/Ar ratio. The results of Laegreid et al. [20] showed that the sputtering yields of the target materials increased with the molecular weight of the working gas. The molecular weight of Ar is much larger than that of C_2_H_2_. Thus, the increase in Si and Ag contents should be related to the increase of the sputtering yields of Si and Ag as Ar ions fraction increased in the working plasma. In addition, compared to the working gas with pure Ar, the existing of C_2_H_2_ in the working gas led to a larger decrease of the sputtering yield of Ag than that of Si. As a result, the content of Ag was always slightly lower than that of Si in all the films regardless of the flow rate ratio. However, the change of C_2_H_2_/Ar ratio had a slight effect on the concentration of O and they were ~15 at. %. This mainly is as a result of the absorption of oxygen when the films expose to ambient air, respectively. Meanwhile, the thickness of the films increased from the 1.0 ± 0.07 μm to 2.0 ± 0.1 μm with the C_2_H_2_/Ar ratio, as shown in Figure 1b, meaning that the film deposition rate increased from 16.7 nm/min to 33.3 nm/min. The high deposition rate was comparable to previous work [21] in which similar precursor gases were used.

**Figure 2:**
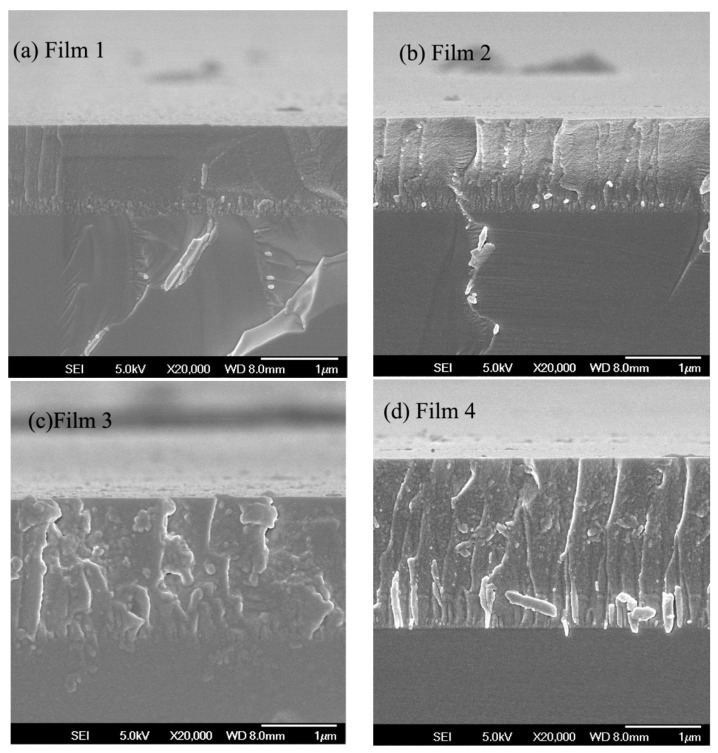
FESEM images of the fractured cross section of the films deposited at different C_2_H_2_/Ar ratios: (**a**) 1/6.0, (**b**) 1/6.5, (**c**) 1/7.0, and (**d**) 1/7.5.

The cross-sectional morphology of the films deposited at different C_2_H_2_/Ar ratios was surveyed by FESEM, as shown in Figure 2. It can be seen that all films were compact and exhibited good adhesion to substrates. More shallow dimples were found on the fractured cross sections of Films 2 and 4, indicating plastic deformations during the preparation of the film for FESEM observation [22]. In addition, Films 3 and 4 were found to contain some large nanoparticles, with no obvious nanoparticles observed on the fractured cross sections of Films 1 and 2.

**Figure 3:**
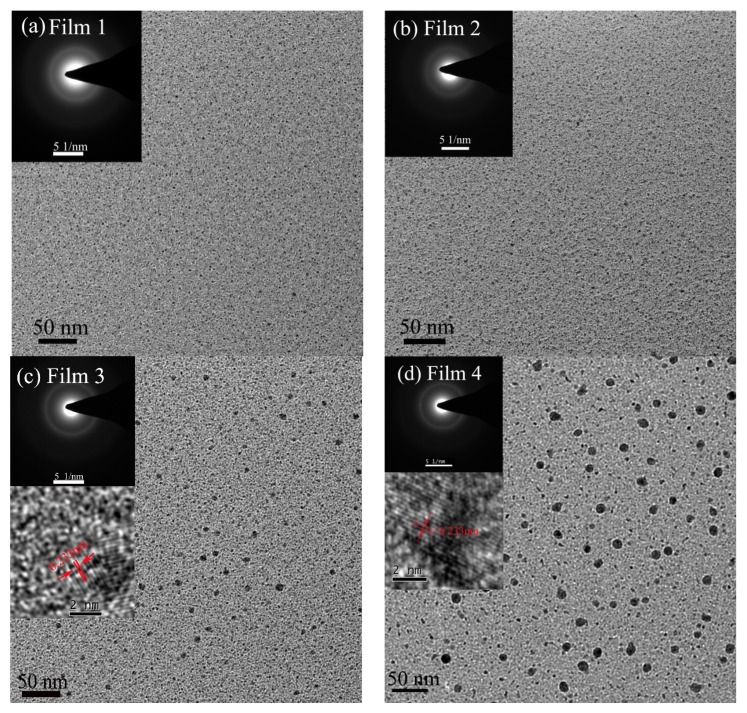
Plane-view HRTEM images of g-C:H:Si:Ag films deposited at C_2_H_2_/Ar ratio of (**a**) 1/6.0, (**b**) 1/6.5, (**c**) 1/7.0, and (**d**) 1/7.5. The inserts in (**a**,**d**) are the selected area X-ray diffraction images of the films, and the lower inserts in (**c**,**d**) are inverse filtered Fast Fourier Transformed (FFT) images of the nanoparticle in the films.

The microstructure of the films was further investigated through high resolution TEM (HRTEM), as shown in Figure 3a–d. The black dots in the figure correspond to nanoparticles. The results of HRTEM further confirmed the nanoparticle-containing microstructure of the films, noting that the average lattice spacing of the particles in Films 3 and 4 was ~2.33 ± 0.02 Å, close to that of Ag (*d*_111_ = 2.35 Å). Si clusters prepared by physical vapor deposition usually exhibited the lattice planes of (111) with the spacing of ~3.1 Å [23,24]. It can therefore be believed that the nanoparticles mainly consist of Ag. Furthermore, particle size in the g-C:H:Si:Ag films gradually increased with C_2_H_2_/Ar ratio decrease. This could be related to the increase of Ag content [25], which was caused by increase of sputtering yields of Ag as the Ar ions increased in the working plasma. Meanwhile, diffusion of the deposited atoms and clusters was enhanced by the increase of Ar ion bombardments during the film’s deposition, and would be favored by the growth of nanoparticles. However, all the films exhibited amorphous features, as indicated by the results of the selected area X-ray diffractions, as shown in the inserts of Figure 3a–d.

### 3.2. Raman Spectra

**Figure 4:**
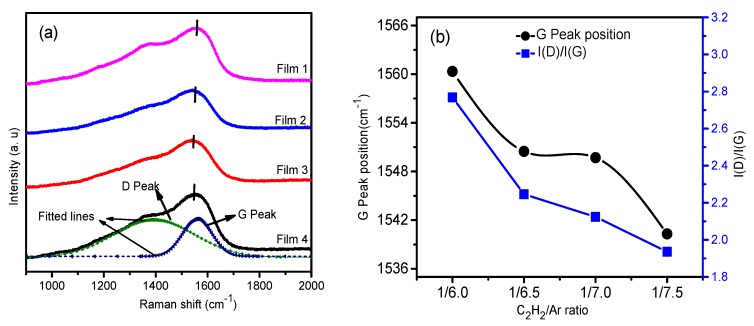
(**a**) Raman spectra at excitation wavelength of 532 nm and (**b**) the fitting parameters of the deposited films.

Raman spectroscopy is extensively used for detecting the bonding structure of carbon atoms in amorphous carbon films since the different carbon bonds always have energy gaps between 0 and 5.5 eV, which match those of Raman scattering. Figure 4a shows the Raman spectra of the films in the range of 900–2000 cm^−1^. The films have typical Raman spectra of amorphous carbon, and the major peak and the shoulder peak of the spectra were found at about 1300 cm^−1^ and 1500 cm^−1^, respectively. The spectrum lines of the films were fitted with Gaussian function to acquire the evolution of chemical bonds. Two fitted peaks, so called G (around 1550 cm^−1^) and D (around 1350 cm^−1^) peaks, were obtained. The former is attributed to the stretching of all pairs of sp^2^ carbon bonds in rings and chains, while the latter depends on the breathing modes of sp^2^ bonds in the rings only [26]. The intensity ratio of D and G peaks, I(D)/I(G) was acquired from the area ratio of the fitted peaks. I(D)/I(G) ratios of the films, ranging from 1.9 to 2.8, are much higher than those of diamond-like carbon films with high sp^3^ hybridization [27]. These data indicate that the bonds of carbon atoms in all the films are mainly sp^2^ hybridization [28]. Thus, it can be speculated that all the films had a feature of graphite-like amorphous structure. Furthermore, as shown in Figure 4b, the G peak of the films shifted toward low frequency with the decrease of C_2_H_2_ fraction in the precursor gas. The intensity ratio of the fitted peaks, I(D)/I(G), also decreased. As suggested by Ferrari et al. [26] and Irmer et al. [29], I(D)/I(G) reduction and the shift toward low frequency of the G peak position were supposed to the results of the increase of the sp^3^/sp^2^ ratio of the carbon atoms. Thus, the sp^3^/sp^2^ ratio of films should increase with the decrease of the fraction of C_2_H_2_ in the precursor gas.

### 3.3. XPS Analyses

**Figure 5:**
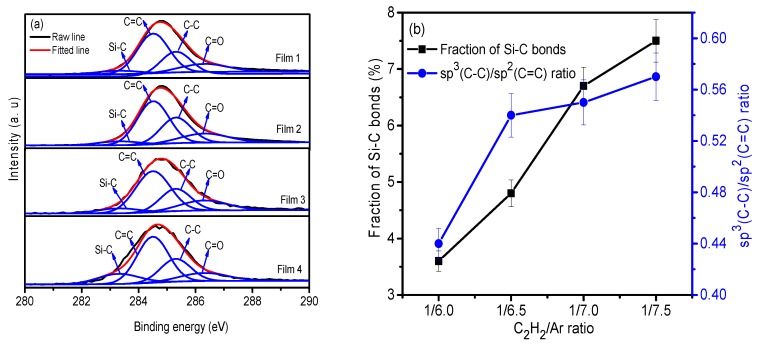
(**a**) The fitted C_1s_ peaks of the deposited films, and (**b**) the fitted results of the C_1s_ XPS peaks of the deposited films.

Further analyses of carbon bonds in films were done via C_1s_ peaks’ deconvolution, as shown in Figure 5. C_1s_ peaks were fitted into four peaks with binding energies of 286.3 ± 0.2 eV, 285.3 ± 0.1 eV, 284.5 ± 0.1 eV, and 283.3 ± 0.2 eV. These were assigned to the bonds of C=O, C–C, C=C, and Si–C, respectively. The fractions of these chemical bonds were calculated from the area of the fitted peaks. The results show that the C=C peak had the largest area in all films, meaning that all films were dominated by the C=C bonds, which is in good agreement with the Raman spectra results. Meanwhile, the Si–C peak area increased with decrease in the C_2_H_2_ fraction, meaning that the Si–C fraction in the films increased due to the increase of Si content in the films, as illustrated in Figure 5b. In addition, the ratio of sp^3^(C–C)/sp^2^(C=C) was found to increase as the C_2_H_2_ fraction decreased.

It has been widely reported that alloying Si into amorphous carbon films can enhance sp^3^ carbon bond formation due to the opening of C=C bonds to form Si–C bonds [18,30], while adding Ag has the opposite effect on sp^3^ carbon bonds [9]. However, the content of Si is higher than that of Ag and increase with the decease of C_2_H_2_ fraction, meaning that the regulation of Si on the carbon bonds is stronger than that of Ag, thus the sp^3^ carbon fraction of the deposited films increases with the decrease of the fraction of C_2_H_2_.

### 3.4. Mechanical and Tribological Properties

**Figure 6:**
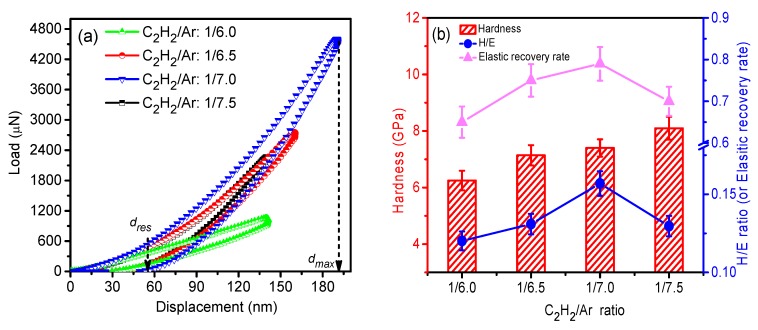
(**a**) Load-displacement curves of the films, and (**b**) hardness, the ratio of hardness and elastic modulus (H/E), and the elastic recovery rate of the deposited films as functions of the C_2_H_2_/Ar ratio.

Figure 6a shows the load-displacement curves of the films prepared in different working atmospheres. The curves of all the films exhibit a relatively low residual displacement, indicating that all the samples have high elasticity. The hardness, elastic modulus, and elastic recovery rate were calculated from the displacement curves, as shown in Figure 6b. It can be seen that the hardness of the films slightly increased with the decrease of C_2_H_2_ fraction. The increase of hardness should be correlated to the increase of Si–C and sp^3^(C–C) bonds which are considered as the major contribution to the hardness of amorphous carbon films containing Si [18,30]. On the other hand, as a results of the softening effect of Ag [9], the hardness of the amorphous carbon films just has a slight increment. Moreover, the film prepared at 1/7.0 has the maximum elastic recovery rate and the largest H/E ratio, which are usually regarded as the important indicators of the tribological performance of the ceramic coatings [31,32,33].

**Figure 7:**
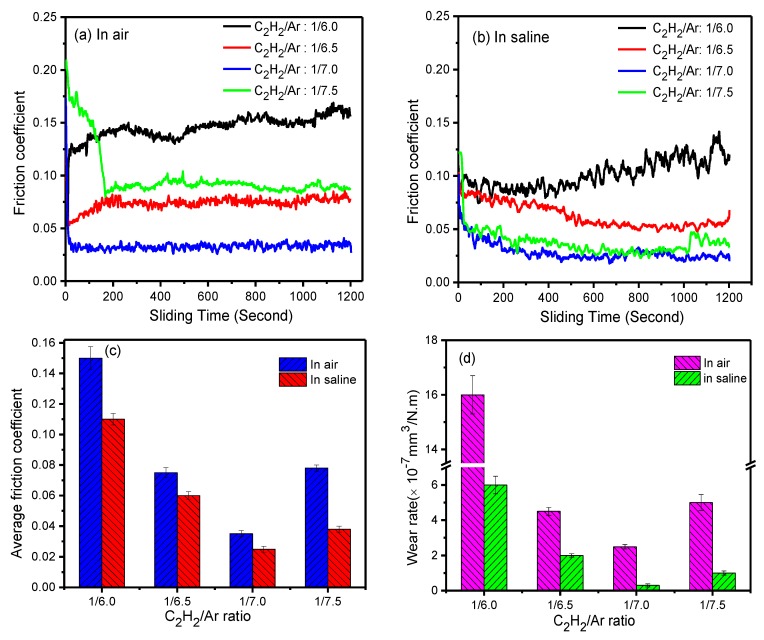
The friction curves obtained in ambient air (**a**), saline solution (**b**), average friction coefficient (**c**), and wear rate (**d**) of the films as functions of the C_2_H_2_/Ar ratio.

The tribological tests were carried out in air ambient and in saline solution (0.9 wt. %) to investigate frictional behaviors of the deposited films. Figure 7a,b show the friction curves of the films in two environments as functions of sliding time. The friction coefficient of the films both in air and in saline solution degrades with the decrease of C_2_H_2_ fraction. The average friction coefficients of the films obtained in saline solution were lower than those in ambient air, as shown in Figure 7c, and an identical feature also occurred to wear rates of the films, as shown in Figure 7d. Moreover, it can be noted that Film 3, prepared at 1/7.0 C_2_H_2_/Ar ratio, had outstanding tribological performance both in air and in saline solution. Especially, its average friction coefficient and wear rate were as low as 0.02 and 3 × 10^−8^ mm^3^/(N·m) in saline solution, respectively.

**Figure 8:**
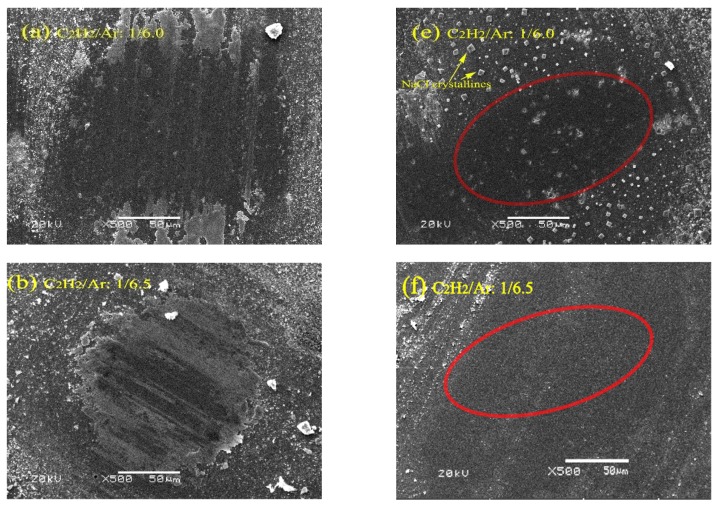

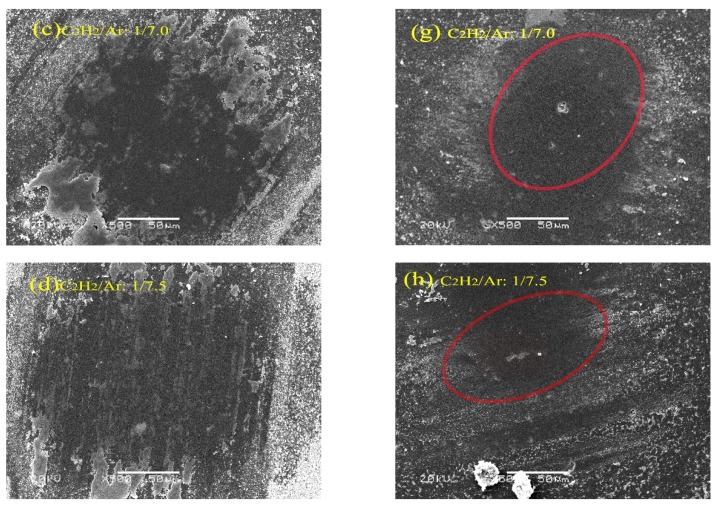
SEM images of the wear scars on the counter balls sliding on the films prepared at different C_2_H_2_/Ar ratio in air (**a**–**d**), and in saline solution (**e**–**h**).

Figure 8 shows the morphologies of wear scars on the counter balls sliding on the films in air and in saline solution. Mechanically, the compact and continuous transfer layer on counter surface is very important for the amorphous carbon films to achieve low friction under dry sliding in air [34,35,36,37]. Some transfer layers can be obviously found on the counter balls sliding in air, as seen in Figure 8a–d. The transfer layer obtained in air from the film prepared at 1/7.0 ratio was a little denser and more continuous than those from other films, and this could be responsible for its better tribological performance in air. On the other hand, there no visible transfer layers were found in the central contact area on the balls sliding in saline solution, as marked by red oval in Figure 8e–h. Typically, in the case of boundary lubrication, some liquid membranes exist in the sliding interface as the films slid in water solution and that can lead to reduction of solid–solid contact. Thus, unlike in the dry sliding, it is usually difficult to form a compact and continuous transfer layer on the ceramic counter balls under boundary lubrication.

**Figure 9:**
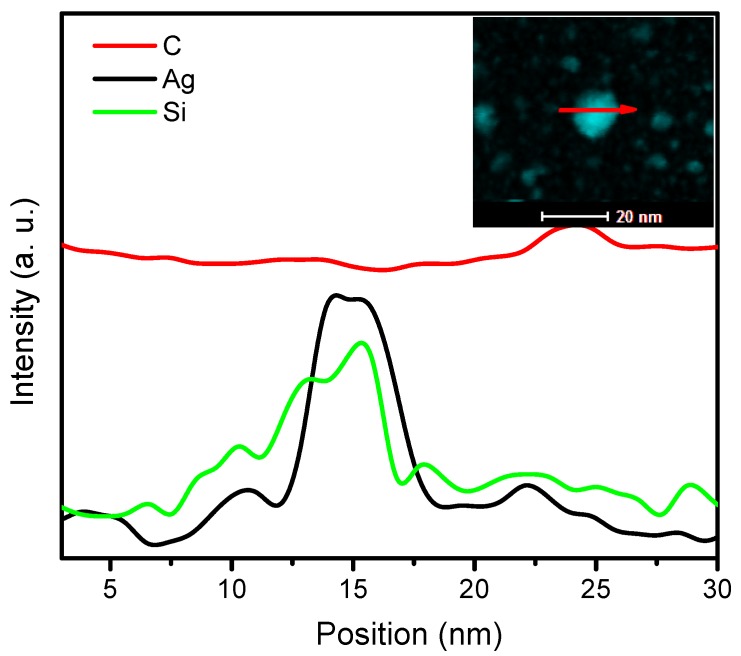
The linear scanning EDS of a nanoparticle in Film 4 (The insert is HAADF-STEM image of Film 4 and the scanning direction is along the red arrow).

Si introduction has significant effects on the boundary lubrication properties of the amorphous carbon films in water solution due to the formation of silica hydrate (SiO_x_(OH)_y_) with low shear strength in the sliding interface, as reported by previous works [17,38,39]. Therefore, decrease in the friction coefficient in saline solution should be mainly related to the increase of the Si content in the films. Due to the high Si content, the friction coefficient of Film 4 had a more notable decrease than that of other films when the testing environment shifted from ambient air to saline solution. Even so, the average friction coefficient of Film 4 was still higher than that of Film 3, though the former contained more Si, as seen in Figure 7c. This could be attributed to the increase in local accumulation of the Si. As shown in Figure 9, Si was found to aggregate in areas where Ag aggregated into particles, which could not be beneficial to the formation and distribution of silica (SiO_x_(OH)_y_) in the sliding interface. In other words, the local accumulation of the Si could not be favorable for the Si composited a-C:H films to achieve a low friction in water solutions though Si content is high.

## 4. Conclusions

The graphite-like carbon composite films containing Si and Ag were prepared by mid-frequency magnetron sputtering deposition in Ar and C_2_H_2_ mixed plasma. The effects of C_2_H_2_/Ar ratio of the working gases on the microstructure and properties of the films were investigated. The results of XPS and Raman spectra show that the fractions of the Si, Ag, and sp^3^ carbon bonds in the g-C:H:Si:Ag films increase with the decrease of the ratio of C_2_H_2_. In addition, Ag was found to aggregate into nanoparticles and their size increased with the films’ silver content. In g-C:H:Si:Ag films with high Si content, silicon was found to accumulate in areas where Ag nanoparticles formed. However, based on the comparative results of friction tests, local accumulation of Si could not be favorable for Si-composited a-C:H films to achieve a low friction in aqueous solutions. Especially, a g-C:H:Si:Ag film prepared at C_2_H_2_/Ar ratio of 1/7.0, showed an ultra-low friction and superior wear resistance both in ambient air and in saline solution.

## Figures and Tables

**Table 1:** The film deposition parameters.

Items	Values
Negative bias voltage on substrates	−200 V
Duty cycle of negative bias voltage	70%
Bias frequency of negative bias voltage	40 kHz
Working pressure	0.5 Pa
Target current	2.0 A
Target voltage	570 V
Substrate temperature	60–65 °C (Without heating)
Deposition duration	60 min
C_2_H_2_/Ar flow rate ratio	Film 1: 1/6.0
	Film 2: 1/6.5
	Film 3: 1/7.0
	Film 4: 1/7.5
